# Cognitive and transcriptomic effects of Epigallocatechin gallate in fetal alcohol spectrum disorders

**DOI:** 10.1038/s41598-025-34576-1

**Published:** 2026-01-02

**Authors:** Anna Ramos-Triguero, Marta Astals-Vizcaino, Elisabet Navarro-Tapia, Melina Vieiros, Adriana Bastons-Compta, Leopoldo Martínez, Vicente Andreu-Fernández, Óscar García-Algar

**Affiliations:** 1 Grup de Recerca Infancia i Entorn (GRIE), Institut d’investigacions Biomèdiques August Pi i Sunyer (IDIBAPS), Barcelona, Spain; 2 Department de Cirurgia i Especialitats Mèdico-Quirúrgiques, Universitat de Barcelona, Barcelona, Spain; 3 Institute for Biomedical Research La Paz (IdiPaz), Madrid, Spain; 4 Department of Neonatology, Hospital Clínic-Maternitat, ICGON, BCNatal, Barcelona, Spain; 5 Faculty of Health Sciences, Valencian International University (VIU), Valencia, Spain; 6 Department of Pediatric Surgery, Hospital Universitario La Paz, Madrid, Spain; 7 Biosanitary Research Institute, Valencian International University (VIU), Valencia, Spain

**Keywords:** Fetal alcohol spectrum disorders (FASD), Epigallocatechin gallate (EGCG), Prenatal alcohol exposure (PAE), Neuroinflammation, Cognitive enhancement, Neuropsychology, Diseases, Neurology, Neuroscience

## Abstract

Prenatal alcohol exposure impacts fetal development, leading to Fetal Alcohol Spectrum Disorders (FASD) characterized by cognitive, behavioral, and physical impairments. This pre-post, open-label, non-randomized pilot study explores Epigallocatechin-3-Gallate (EGCG), a potent antioxidant and neuronal plasticity modulator, as a therapeutic intervention for FASD improvement. In a 12-month pilot study, patients with 40 FASD (mean age 10 ± 3 years) received 9 mg/kg/day of EGCG, with outcomes assessed through RNA sequencing and neurocognitive evaluations (WISC-IV, CBCL 6–18, and NEPSY-II). Reduced levels of oxidative stress were observed after 6 and 12 months of treatment with EGCG. Significant neurocognitive improvements were shown after one year of treatment in Perceptual Reasoning Index (PRI) and Working Memory Index (WMI) using the WISC-IV test. CBCL test revealed an improvement in aggressive behavior scores after EGCG treatment. NEPSY-II assessment showed improvements in face memory and delayed face memory. Interestingly, no significant improvements were observed in intelligence quotient, attention, anxiety, or depression, which affect a proportion of individuals diagnosed with FASD. Additionally, EGCG modulates molecular pathways associated with neuroinflammation, immune response, and neurogenesis. This study highlights EGCG as a potential therapeutic candidate to ameliorate cognitive and behavioral deficits in children affected by FASD, revealing potential pathways and biomarkers that contribute to these improvements.

*ClinicalTrials.gov identifier: *NCT02558933 (registered 22 September 2015).

**Supplementary Information:**

The online version contains supplementary material available at 10.1038/s41598-025-34576-1.

## Introduction

Alcohol consumption during pregnancy produces significant risks to fetal development, leading to neurodevelopmental disorders known as Fetal Alcohol Spectrum Disorder (FASD). Prenatal alcohol exposure (PAE) damage depends on pregnancy stage, dose, consumption pattern, and genetic susceptibility^1^. FASD includes deficits in executive function, learning, memory, general cognition, language, and social skills^2^.

Global prevalence of FASD is 7.7 per 1000 population, with the highest prevalence (19.8 per 1000) in European Regions^3^. However, PAE and FASD are notably higher in Eastern European Countries (EEC) orphanages, with prevalences ranging from 15% to 68%^4,5^. In addition, among adopted children from EEC, at least 50% exhibit FASD-related symptoms^6^.

Although molecular mechanisms of FASD are not fully understood, PAE produces high levels of reactive oxygen species (ROS), causing oxidative stress and disrupting critical homeostatic pathways^7^. PAE leads to microcephaly and brain architecture and connectivity alterations^8^. Neuroinflammation mediated by microglia activation plays a key role in these effects, with pathways involving MAPK and Notch signaling^9^. Mitochondrial dysfunction has also emerged as a central contributor to neurodevelopmental impairment. Deficits in ATP production and increased mitochondrial ROS generation are reported across neurodevelopmental disorders^10^ and have recently been described in FASD models, where prenatal alcohol exposure alters mitochondrial morphology, respiratory chain activity, and redox balance^11,12^. These mitochondrial deficits exacerbate neuronal vulnerability and may underlie part of the cognitive and behavioral phenotype observed in FASD.

Transcriptomic research is crucial for a comprehensive analysis of gene expression patterns in FASD. Limited transcriptomic studies have reported dysregulated pathways related to cell cycle, neurodevelopment, neuroinflammation, apoptosis, and immune function^9,13,14^. Additionally, there have been improvements in the investigation of genetic polymorphisms^15^ and the use of artificial intelligence for FASD diagnosis^16^. However, current research is limited by small sample sizes, lack of longitudinal studies, and variability in diagnostic criteria.

Prevention of FASD relies solely on avoiding alcohol during pregnancy, as no treatments address its neurodevelopmental impairments. Pharmacological treatments target symptoms such as hyperactivity, attention problems, aggressiveness, and anxiety^17,18^. Efforts to enhance neuronal plasticity, synaptic transmission, and reduce oxidative stress are crucial for long-term cognitive and behavioral improvements. Natural antioxidants, such as epigallocatechin-3-gallate (EGCG), showed potential to address neuronal plasticity dysfunction in Alzheimer’s disease and Down syndrome (DS)^19–21^. EGCG targets the root of neurodevelopmental impairments by enhancing neural communication and reducing ROS. In a randomized clinical trial, De la Torre et al. demonstrated that EGCG combined with cognitive training improved visual recognition memory, inhibitory control, and adaptive behavior in individuals with Down syndrome^21^. Given that FASD and DS share similar dysmorphologies, such as craniofacial defects and DYRK1A and RCAN1 genes dysregulation^22^, EGCG could potentially improve cognitive performance in FASD. Additionally, EGCG has recently been shown to improve mitochondrial function in vivo. In young children with Down syndrome, decaffeinated EGCG supplementation restored mitochondrial complex I and complex V activity in peripheral cells^23^, suggesting that EGCG can modulate bioenergetic deficits relevant to neurodevelopmental disorders, including those observed in FASD.

EGCG, the major polyphenol in green tea, reduces oxidative stress by scavenging radicals, inhibiting lipid-peroxidation, and restoring antioxidant levels^24^. Green tea has exhibited protective effects against neuroinflammation, immune system, and neural plasticity^25^.

The objective of this study is to analyze how this antioxidant modifies the transcriptional pattern of global expression in blood samples from patients with FASD and thereby improve cognitive performance, helping to unravel the molecular mechanisms that could contribute to early diagnosis and treatment of this disorder.

## Results

**Fig. 1:**
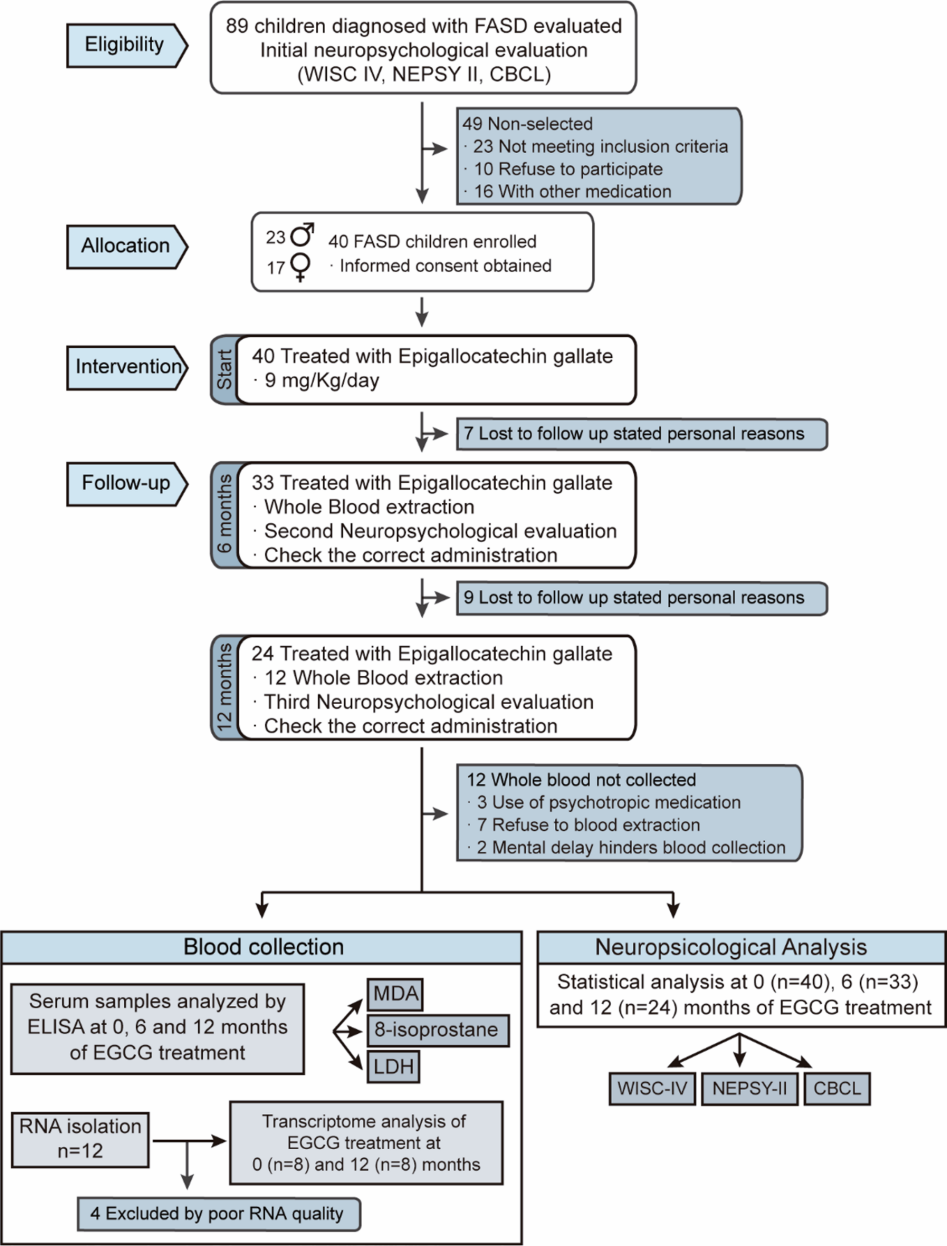
CONSORT Diagram. Flowchart of the study in children with FASD during 12 months.

The study initially enrolled 89 patients, with 49 exclusions: 23 did not meet inclusion criteria, 10 declined participation, and 16 were taking interfering medications. Of the remaining 40 patients (mean age 10 ± 3 years), 18 met diagnostic criteria for FAS and 22 for partial FAS (pFAS). During follow-up, 7 children dropped out before the 6-month visit, leaving 33 children who completed the 6-month evaluation. Between the 6- and 12-month visits, an additional 9 children were lost to follow-up, resulting in 24 children completing the intervention (Fig. 1; Table 1).

**Table 1:** Distribution by sex, mean height, weight, BMI and age of the FASD participants.

Gender ♂/♀	*N* (%)	Diagnosis		Height, cm ± SD	Weight, kg ± SD	BMI ± SD	Age, years ± SD	Min, years	Max, years
		FAS	pFAS						
Male	23 (57.5)	8	15	139.0 ± 15.6	32.1 ± 10.3	16.1 ± 1.8	10 ± 2	7	16
Female	17 (42.5)	10	7	130.9 ± 18.0	30.6 ± 12.0	17.1 ± 2.9	10 ± 3	5	16
Total	40 (100)	18	22	135.5 ± 16.9	31.4 ± 10.9	16.5 ± 2.4	10 ± 3	5	16

To evaluate EGCG as a therapeutic candidate for FASD, neurocognitive evaluation was performed at baseline (*n* = 40), 6 (*n* = 33), and 12 months (*n* = 24). Oxidative stress status was assessed at baseline and 6 and 12 months. At 12 months, 24 patients completed the treatment. Nevertheless, 12 blood samples were not collected due to pain and non-compliance in children. Of the 12 collected, 4 were excluded for not meeting RNAseq quality criteria.

### EGCG improves neurocognitive and behavioral scores in FASD

**Fig. 2:**
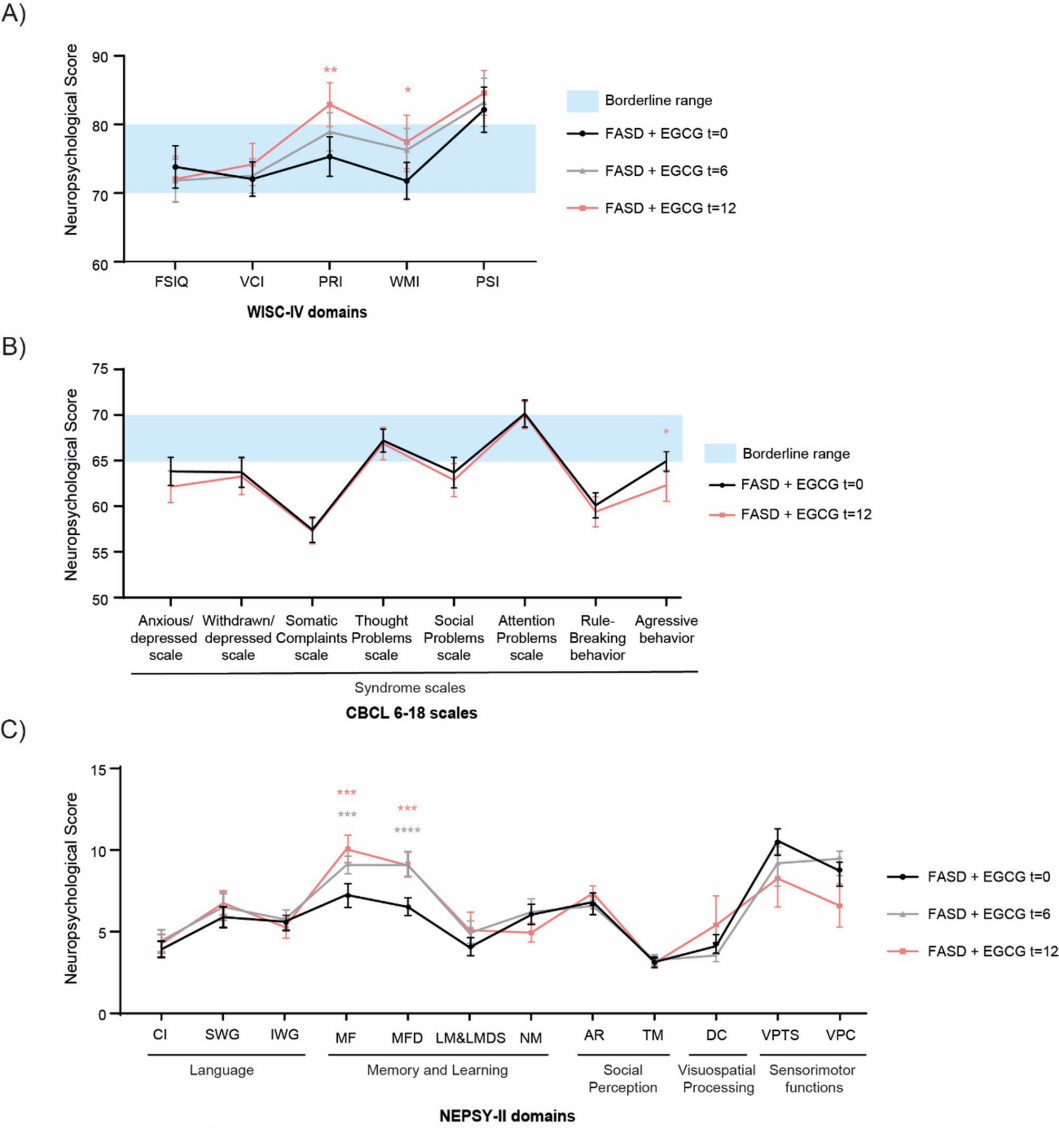
Neurocognitive assessment. Comparison of neuropsychological profiles of FASD at baseline, 6 and 12 months after EGCG treatment. (**A**) WISC-IV test. (**B**) CBCL 6–18 test. (**C**) NEPSY-II test. Affect Recognition (AR); Comprehension of Instructions (CI); Design Copying (DC); Full-Scale IQ (FSIQ); Initial Word Generation (IWG); List Memory & List Memory Delayed (LM&LMD); Memory for Faces (MF); Memory for Faces Delayed (MFD); Narrative Memory (NM); Perceptual Reasoning Index (PRI); Processing Speed Index (PSI); Semantic Word Generation (SWG); Theory of Mind (TM); Verbal Comprehension Index (VCI); Visuomotor Precision Combined (VPC); Visuomotor Precision Time (VPTS); Working Memory Index (WMI). Data are represented as mean ± SEM of each score. Black line shows distribution of FASD at baseline, gray line shows distribution after 6 months and red line shows distribution after 12 months of EGCG treatment. Blue box indicates borderline range (when applicable). For WISC: 70–79; for CBCL: 60–63; NEPSY-II does not define a borderline range — scores are classified as well below, below, average, above, or well above expected. Significance indicated for comparisons between FASD patients at baseline and after 12 months of treatment. ****, *p* < 0.001, ***, *p* < 0.005, **, *p* < 0.01, *, *p* < 0.05.

To assess cognitive improvement, WISC-IV scale was administered at baseline, 6, and 12 months after EGCG treatment (Figs. 2a, Supplementary Table S1). In this test, higher scores reflect better cognitive performance, with values above the *borderline range* representing the average/normal population. 12 months results showed improvement in PRI (*p* = 0.002) and WMI (*p* = 0.04) compared to baseline, with PRI scores moving out of the borderline range, while WMI scores reached the upper end of the borderline threshold.

CBCL neurocognitive assessment compared baseline to 12-month results (Figs. 2b, Supplementary Figure S1, Supplementary Table S2). In this case, higher scores indicate greater behavioral problems, with values in the borderline/clinical range reflecting pathology. After 12 months, a significant reduction in Aggressive Behavior score (*p* = 0.03) was observed, with scores stabilizing at the borderline threshold.

NEPSY-II evaluated neuropsychological performance pre- and post-intervention (Fig. 2c, Supplementary Table S3). For this scale, higher scores represent better performance, with higher values reflecting closer alignment to expected developmental levels. Significant improvements after 6 and 12 months were obtained for Memory for Faces (MF, *p =* 0.02, *p* = 0.005), Memory for Faces Delayed (MFD, *p* < 0.003, *p* = 0.006*).*

### EGCG reduces oxidative stress in FASD

**Fig. 3:**
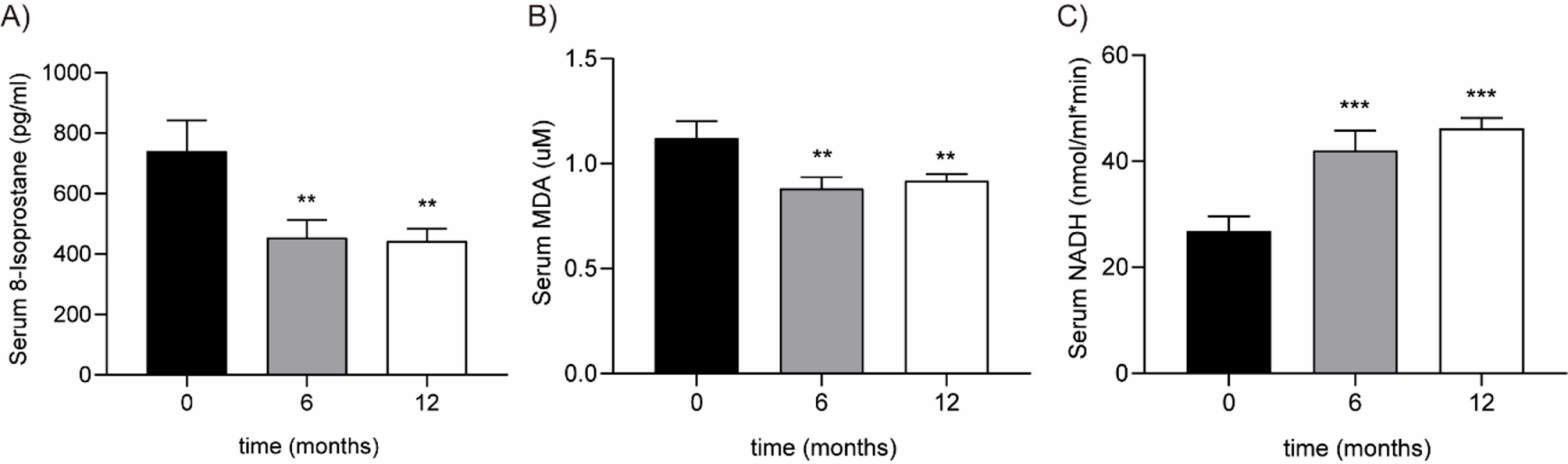
Levels of oxidative stress biomarkers at baseline, 6 and 12 months after EGCG treatment. Serum concentrations of 8-isoprostane (**A**), malondialdehyde (**B**) and NADH+ (**C**) in FASD children were compared. Data are presented as mean ± SEM. Comparisons across time points were performed using paired statistical tests (Wilcoxon signed-rank test). * *p* < 0.05; ** *p* < 0.01; *** *p* < 0.001.

In 33 FASD patients (19 males, 14 females) at baseline and after 6 months of treatment, oxidative stress was measured by quantifying MDA and 8-isoprostane. MDA, a byproduct of fatty acid oxidation^26^, and 8-Isoprostane, ROS-induced metabolite^27^, decreased by 39% (*p* = 0.0017) and 21% (*p* = 0.003), respectively (Figs. 3a-b). These reductions were maintained at 12 months, where both 8-isoprostane and MDA levels remained significantly lower than baseline, indicating sustained attenuation of lipid peroxidation.

LDH activity, evaluated by NADH/mL*min (Fig. 3c), measured catalyzation of NAD + to NADH. In mitochondrial respiration, NADH donates electrons to Complex I, producing NAD + and O_2_^−^and subsequently H_2_O_2_ in Complex III, increasing ROS production. A 57% increase in NADH after 6 months, this trend was also shown at 12 months, indicating a significant reduction of oxidative stress (*p* < 0.0001).

These results showed a reduction in oxidative stress markers after 6 and 12 months of EGCG treatment.

### EGCG modifies FASD transcriptomic profile

RNAseq of blood samples from FASD patients revealed the transcriptomic profile of FASD patients and EGCG treatment effect. PCA confirmed no outliers and clear differences (Supplementary Figure S2) between baseline and 12 months of EGCG treatment.

**Fig. 4:**
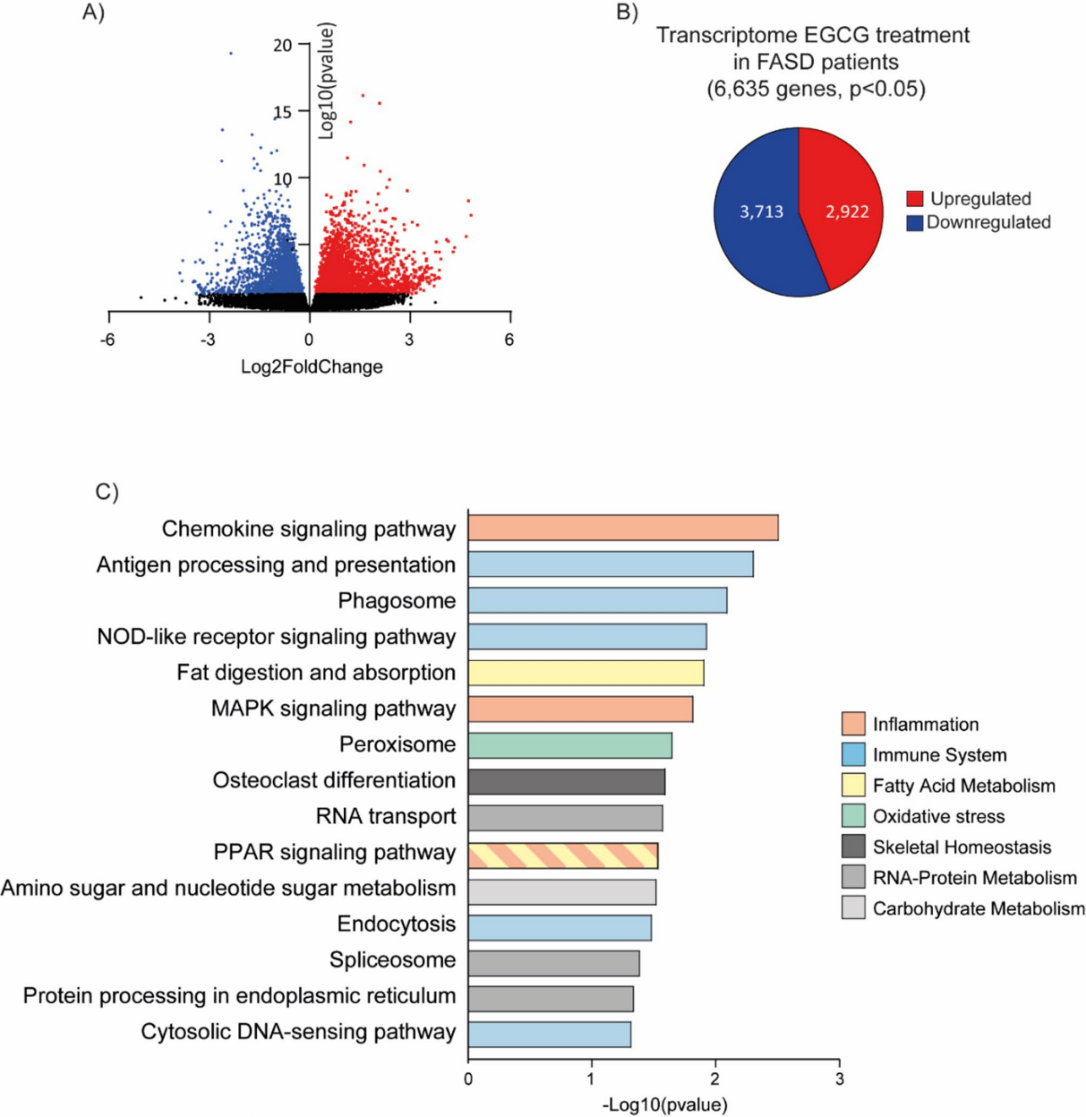
Transcriptomic analysis in FASD patients after EGCG treatment. (**A**) Volcano plot indicates effect size (Log2FoldChange) and significance (Log10pvalue) for each transcript comparing at baseline and after EGCG treatment. Red dots are up-regulated significant genes and blue dots are down-regulated significant genes. (**B**) Circular Diagram indicates 6635 differentially expressed genes in the transcriptomic analysis. (**C**) KEGG pathways were observed with significant genes comparing before and after EGCG treatment.

62,712 expressed genes were detected by comparing 8 FASD patients (4 male, 4 female) at baseline and after 12 months. 6,635 differentially expressed genes (DEG) were identified after EGCG treatment (Fig. 4a), comprising 2,922 downregulated and 3,713 upregulated (Fig. 4b).

KEGG analysis^28–30^ was performed among genes significantly expressed after EGCG treatment. Enriched pathways can be grouped based on their function (Fig. 4c, Supplementary Table S4), showing significant differences in inflammation-related pathways, such as chemokine signaling, MAPK, PPAR, and immune system pathways, including antigen processing and presentation, phagosome, NOD-like receptor, endocytosis and cytosolic DNA-sensing pathway. Additionally, fatty acid metabolism, encompassing fat digestion and absorption and oxidative stress pathways, such as peroxisome. Other pathways involved were related to skeletal homeostasis, RNA-protein, and carbohydrate metabolism.

**Fig. 5:**
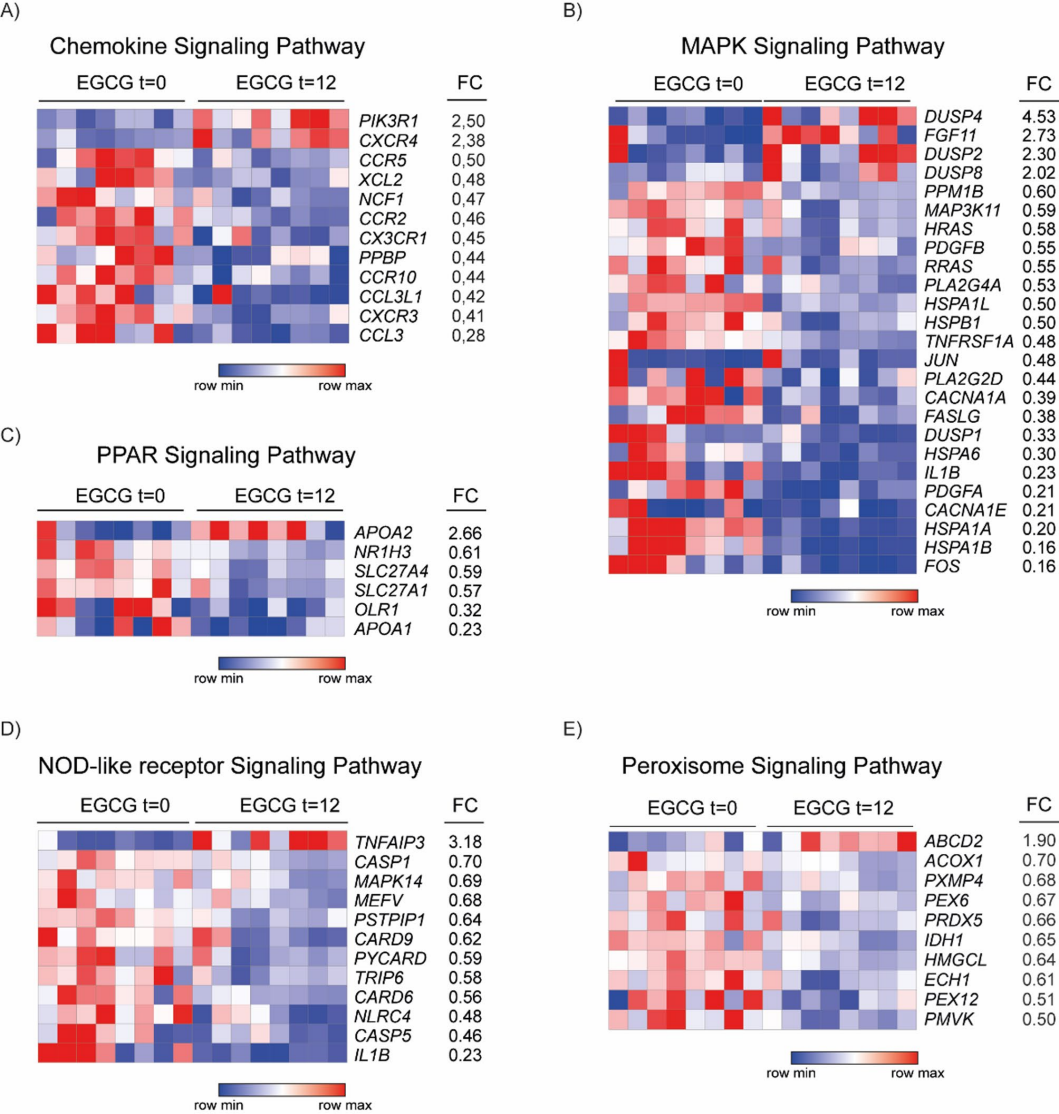
Heatmap of enriched pathways after treatment. Heatmap shows DEGs genes comparing at baseline and after EGCG treatment. Analysis was performed with FDR < 0.05, FoldChange (FC) > 1.8 and < 0.7. Downregulated genes are indicated in blue and upregulated in red.

Pathways relevant to FASD pathogenesis and EGCG treatment were evaluated. Inflammation-related pathways were analyzed, due to the impairment during brain development^31^, as chemokine, MAPK, and PPAR signaling. KEGG analysis highlighted chemokine signaling as the most enriched pathway after EGCG treatment (Fig. 4c). The identified genes are shown in the Heatmap (Fig. 5a), including PI3KR1 and CXCR4 as upregulated genes, and CCR5, XCL2, NCF1, CCR2, CX3CR1, PPNP, CCR10, CCL3L1, CXCR3, and CCL3 as downregulated genes. CXCR3, CX3CR1, and CCR2 genes were selected for validation as they play an important role in PAE, regulating activation of microglia^31^.

Similarly, MAPK pathway was previously observed to be activated in mouse FASD model transcriptome analysis^9^. Our analysis identified DUSP4, FGF11, DUSP2 and DUSP8 as upregulated genes (Fig. 5b), while HSPB1, TNFRSK1A, JUN, PLA2G2D, CACNA1A, FASLG, DUSP1, HSPA6, IL1B, PDGFA, CACNA1E, HSPA1A, HSPA1B and FOS genes were downregulated. For RT-qPCR, we selected DUSP4 and FGF11 as the most significantly upregulated genes and FOS as a principal downstream effector of MAPK pathway and the most downregulated gene.

PPAR signaling controls the transcription of target genes related to immune response, lipid metabolism, oxidative stress, and inflammation^32^. RNAseq analysis identified APOA2 as an upregulated gene and NR1H3, SLC27A4, SLC27A1, OLR1, and APOA1 as downregulated genes (Fig. 5c), which are involved in lipid metabolism, oxidative stress, and inflammation regulation^32,33^. OLR1 was selected for RT-qPCR validation as a direct transcriptional target of PPARγ^34^ and has previously been described to promote microglial migration activation^35^ and may improve cognition in FASD patients.

PAE activates the immune system through TLR and NLR receptors in CNS^36^. Therefore, we focused on NOD-like receptor pathway in this study. Antigen processing and presentation, phagosome, endocytosis, and cytosolic DNA-sensing pathways were not selected for the research because blood sampling may potentially affect the high amount of RNA related to immunoglobulins and immune system cells. Our RNAseq analysis revealed an upregulation of TNFAIP3 gene, whereas CASP1, CASP5, MAPK14, MEFV, PSTPIP1, CARD6, CARD9, PYCARD, TRIP6, NLRC4, and IL1B genes were downregulated (Fig. 5d). For RT-qPCR validation, TNFAIP3 and CASP5 were chosen as the most up- and downregulated genes, respectively, and are involved in NF-kB and inflammasome^37–39^.

Peroxisome signaling is implicated in fatty acid oxidation^40^, oxidative stress, and ROS production^40^, which causes neuronal apoptosis in FASD^41^. The results showed that the peroxisome pathway was mainly downregulated after EGCG treatment in FASD patients, with ABCD2 as an upregulated gene and ACOX1, PXMP4, PEX6, PRDX5, IDH1, HMGCL, ECH1, PEX12, PMVK as downregulated genes (Fig. 5e). ABCD2 and PMVK were selected for validation as the most up- and downregulated genes, respectively.

Pathways related to osteoclast differentiation, RNA transport, spliceosome, protein processing in endoplasmic reticulum, and amino sugar and nucleotide sugar metabolism were not selected for RNAseq validation due to their unclear impact on FASD neurodevelopmental pathogenesis.

**Fig. 6:**
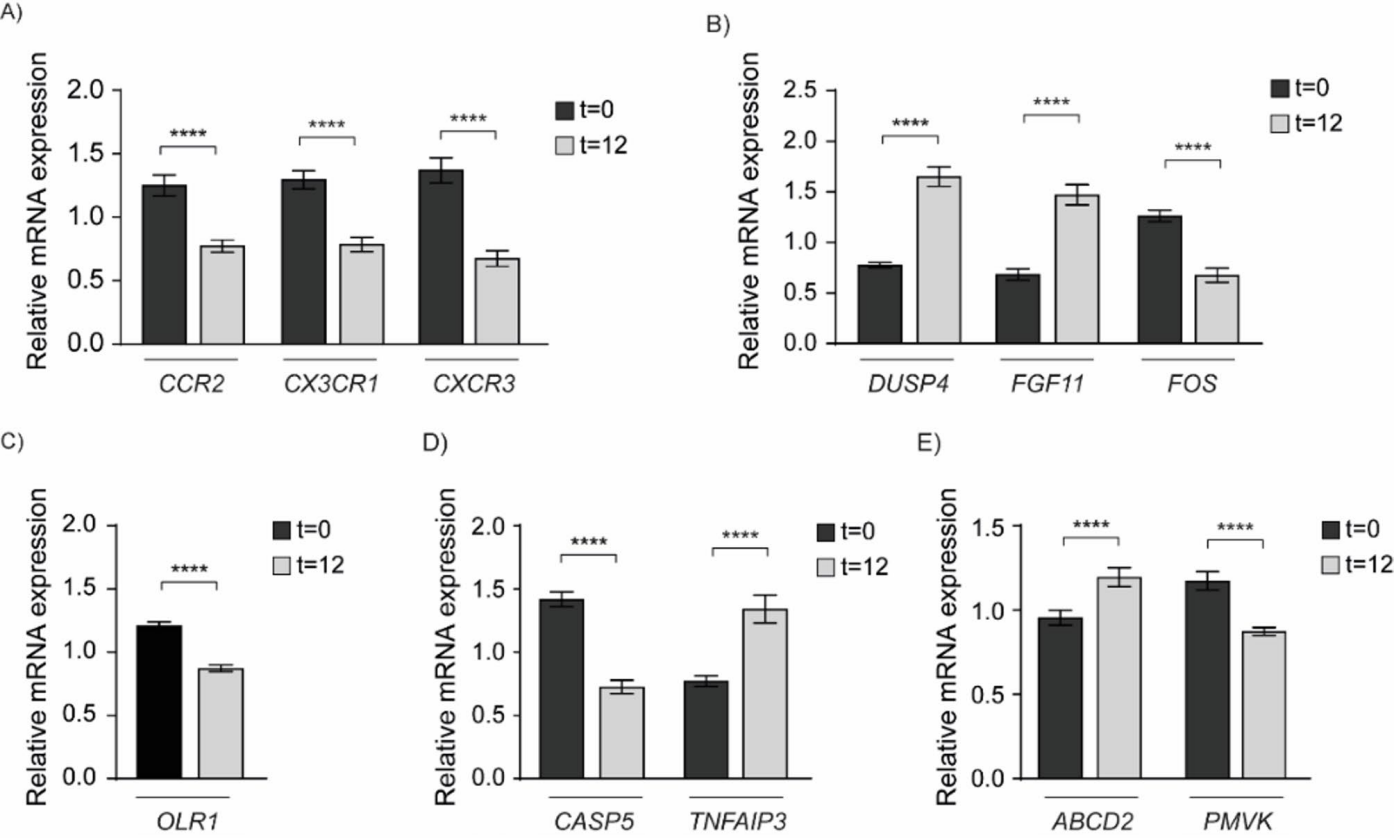
RT-qPCR validation of RNAseq results. Validation of 11 DEGs identified in FASD children before and after EGCG treatment. Relative expression levels of each gene were calculated using the 2-ΔΔCt method. Data are represented as mean ± SEM. ****p < 0.001.

After RT-qPCR, our results showed that gene expression in chemokine pathway of CCR2, CX3CR1, CXCR3 were significantly downregulated after 12 months of treatment (Fig. 6a). In MAPK pathway (Fig. 6b), statistical analysis showed that DUSP4 and FGF11 were upregulated and FOS gene was downregulated after treatment. In PPAR pathway (Fig. 6c), OLR1 was significantly downregulated after EGCG treatment, while the expression levels of CASP5 and TNFAIP3, related to the NOD-like receptor pathway, showed significant down- and upregulation, respectively (Fig. 6d). Finally, in the peroxisome pathway, ABCD2 showed a significant upregulation, whereas PMVK showed the opposite behavior (Fig. 6e). All these results confirmed the findings obtained by RNAseq analysis.

### Safety outcome

The safety and tolerability of the product were good, with no clinically relevant biochemical alterations observed during the study period. No significant differences were detected in glucose levels (*p* = 0.417), AST (*p* = 0.913) or cholesterol (*p* = 0.715) between baseline and twelve months. ALT showed a small increase that did not reach statistical significance (*p* = 0.068). Triglycerides showed a significant decrease over time (*p* = 0.0036), with a decrease of 36 points by the end of the study (Supplementary Figure S5).

## Discussion

This study has evaluated the therapeutic potential of EGCG to improve cognitive and behavioral performance in FASD, showing significant improvements in cognitive and behavioral variables. Furthermore, EGCG induced significant changes in the transcriptomic profiles, modulating key pathways related to CNS function, oxidative stress, anti-inflammatory response, and immune system regulation.

Levels of MDA, 8-isoprostane, and LDH biomarkers after EGCG treatment demonstrated that this antioxidant significantly reduced oxidative stress in FASD, confirming previous observations in other pathologies^42,43^. Moreover, molecular changes observed after EGCG treatment correspond to its documented health benefits, including anti-inflammatory and anti-oxidative properties^44^ by suppressing NLRP3 inflammasome^45^ and modulating the expression of MAPK^46^, PPARγ^47^ and NLR family^48^.

Previous studies highlighted that EGCG could prevent embryonic damage caused by PAE, including growth restriction, neurodevelopmental maturation, and differentiation^25^. Our study has demonstrated significant improvements in intellectual functioning area (WISC-IV) in FASD, including perceptual reasoning, understanding visual information, and non-verbal reasoning. Additionally, working memory, which involves memorization and manipulation of new information, improved in FASD after one year of treatment with EGCG. Executive functioning (NEPSY-II) improved significantly in the ability for face memory. These results are associated with motor plus visual-perceptual skills. Results obtained in problem behavior area (CBCL 6–18) showed a significant decrease in aggressive behavior, one of the main problems in FASD^49^. Our findings are consistent with previous studies which observed improved memory and executive function after EGCG treatment in middle-aged and elderly subjects^50^, DS^21^ and Alzheimer^19^. Similar results were observed in studies of choline supplementation in FASD cohorts. Wozniak et al. examined cognitive variables and demonstrated an increased capacity for long-delayed memory in FASD^51^. Consistent with our results, Nguyen et al. documented an alleviation in memory deficits and an improvement in executive function. However, in line with our findings, their investigations also did not reveal favorable results on measures of attention deficit^52^.

Our transcriptomic analysis provided valuable insights into molecular mechanisms underlying the therapeutic effects of EGCG in FASD. Previous transcriptomic studies in FASD performed in animal models discovered dysregulated pathways related to neurodevelopment, inflammation, and immune function pathways^9,13,53^. Our study was conducted using blood samples from children with FASD, underscoring that human pediatric samples offer a direct connection to clinical practice, making our findings particularly relevant for translational research and potential therapeutic applications.

Enriched pathways were linked to pro-inflammatory response downregulation after EGCG treatment, as chemokine, MAPK, and PPAR pathways. Chemokines released by neurons, microglia, or astrocytes play a crucial role in CNS^54^. According to RT-qPCR results, CXCR3, CX3CR1, and CCR2 expression levels were significantly downregulated after EGCG treatment. These molecules essential for immune cell activation, are associated with neurodegeneration and inflammation^55–57^. Our results indicate that EGCG reduces pro-inflammatory chemokines, as previously confirmed^58–60^.

EGCG inhibits MAPK activation by decreasing ROS^61^ and ERK activity^62^, also implicated in neuroinflammatory processes^63^. Chemokines activate MAPK and PI3K pathways through CXCR3, CX3CR1, and CCR2^64–67^. RT-qPCR showed upregulation of DUSP4 and FGF11 and downregulation of FOS after EGCG treatment. Neuronal DUSP4 overexpression is associated with a decrease in cytokines, chemokines, and IFNγ, reducing neuroinflammation^68^. Although its function in humans is not fully understood, FGF11 mouse homolog is associated with CNS development^69^ and might promote neural development in humans. Moreover, FOS, a downstream target of the MAPK cascade, activates AP-1, a modulator in inflammatory diseases^70^. The specific roles of DUSP4, which negatively regulates the MAPK pathway^71^ and induces FOS reduction, confirm that MAPK downregulation could have a potential role in dampening inflammation after EGCG treatment in FASD.

PPAR nuclear receptors regulate genes involved in immunity, lipid metabolism, oxidative stress, and inflammation^32^. Our findings indicate that PPAR signaling was downregulated after EGCG treatment. RT-qPCR confirmed OLR1 downregulation, a direct PPARγ target^34^. Studies revealed that OLR1 and CCL2 promote neuroinflammation through microglial activation, and their expression is reduced after anti-inflammatory treatment with OPC scFvD3-1^35^. In the same line, our results indicated downregulation of OLR1 and CCR2 expression, confirming the anti-inflammatory effect of EGCG.

NLR signaling also plays role in inflammatory and immune-related pathways. Studies showed that alcohol stimulates astrocytes and microglia activity through the activation of TLR and NLR, resulting in activation of NF-kB, MAPK signaling, and cytokines^72^. Our results showed TNFAIP3 upregulation and CASP5 downregulation. TNFAIP3 acts as a negative feedback regulator of NF-kB, NLR, and apoptosis cascades^73^. CASP5 is a caspase involved in inflammation and apoptosis^39^, and an NLR target^38^. Our findings suggest that EGCG treatment may reduce inflammation and apoptosis through NLR signaling, inducing TNFAIP3 upregulation and observing CASP5 downregulation by reducing NF-kB activation and pro-inflammatory cytokine release.

EGCG can regulate peroxisome-signaling, responsible for fatty acid oxidation and ROS metabolism^40^. ABCD2, which was significantly upregulated, is involved in fatty acid metabolism and peroxisome biogenesis^74^. Its downregulation is associated with proinflammatory cytokines^75^ and oxidative stress^76^. PMVK, significantly downregulated after EGCG treatment, is involved in the mevalonate pathway^77^, which can induce inflammatory and immune responses^78^. Therefore, EGCG increases ABCD2 and reduces PMVK expression, which may modulate oxidative stress and inflammatory response dysregulated in FASD.

Although canonical mitochondrial pathways (e.g., oxidative phosphorylation or TCA cycle) did not emerge as significantly enriched in our analysis, PPAR signaling and peroxisome-related pathways are tightly interconnected with mitochondrial function. PPAR activation regulates mitochondrial β-oxidation and biogenesis through PGC-1α co-activation, shaping cellular energy metabolism and ROS homeostasis^79^. Likewise, peroxisomes engage in extensive metabolic crosstalk with mitochondria, coordinating lipid catabolism and reactive oxygen species detoxification, and alterations in peroxisomal activity can directly impact mitochondrial performance^80^. Therefore, the modulation of PPAR signaling and peroxisomal genes observed after EGCG treatment may reflect indirect regulatory effects on mitochondrial-associated metabolic processes.

Our study found that EGCG improved intellectual, executive, and behavioral outcomes in children with FASD. These results may be attributed to EGCG’s regulation of genes and pathways that promote synaptic plasticity, memory, emotional processing, and limbic system, such as MAPK-pathway^81,82^, which has also been implicated in aggressive behavior, social anxiety, and social defeat^83^. Additionally, PPARγ regulation promotes axonal growth and myelination, supporting cognitive function^84^. EGCG peroxisome may interact with BDNF-TrkB in the adult brain^85^, contributing to memory, synaptic plasticity, and neurogenesis^86^. Additionally, DUSP4 family has been studied for their activity in neuronal differentiation^87^, embryonic stem cell neurogenesis^88^, axon development^89^ and neuroprotection^90^. FGF family plays crucial role in adult neural proliferation and regenerative processes^91^ and FGF11 plays a role in mouse limb development^69^. Our findings also support that FontUp is safe and well tolerated, in line with the results of the PERSEUS study conducted in children aged 6 to 12 years with Down syndrome^20^. While our study provides preliminary insights into the potential therapeutic effects of EGCG in FASD, further research is required to clarify the mechanisms through which EGCG may influence cognition and behavior in humans. In a FASD-like mouse model, our group showed that EGCG restored markers of neuronal maturation (NeuN, GFAP), enhanced synaptic plasticity signatures (BDNF, DYRK1A), and improved learning and memory performance in Rotarod, T-maze and Morris water maze tests^92^. All these results suggest that EGCG has the potential to modulate neuronal development and synaptic plasticity. However, additional mechanistic studies in humans are needed to substantiate this hypothesis.

## Conclusions

This study demonstrates the therapeutic potential of EGCG to improve some cognitive and behavioral variables, including perceptual reasoning, memory, and to reduce aggressive behavior in FASD patients. Interestingly, EGCG did not differentially improve intelligence quotient, anxiety, or depression, which are domains particularly vulnerable in FASD patients. Transcriptomic analysis revealed changes in critical pathways related to anti-inflammatory responses, immune system, and antioxidant response. Our findings provide, for the first time, evidence of the ability of EGCG-based treatments to induce changes at the molecular level that translate into improved cognitive performance in this population, potentially improving the quality of life of patients and their families.

### Limitations of the study

EGCG shows promise as a therapeutic candidate for improving cognition and behavior in FASD. However, some limitations should be considered. The age range of 5 to 16 years, and the lack of sex-based analysis, may introduce biases related to childhood and adolescent growth. However, our pre-post design allows individualized assessments by comparing each participant to their baseline, reducing biases associated with inter-individual variability. The sample size and the reduced number of participants retained at 12 months may limit the statistical power and the ability to detect modest treatment effects. People who dropped out of the trial may have caused an attrition bias, introducing a bias into the estimated effect. CBCL scales were assessed at baseline and after 12 months to minimize participant burden and ensure higher compliance, limiting the impact that 6-month assessments might have on increasing participant dropout due to fatigue, despite the potential loss of information on behavioral change midway through the study. Another limitation is the open-label design, which may have introduced expectation bias in parent-reported outcomes such as the CBCL. Nonetheless, the consistency of improvements across objective cognitive measures (WISC-IV, NEPSY-II) and molecular biomarkers supports the robustness of our findings. Future randomized, double-blind studies are warranted to confirm these results. Although oxidative stress biomarkers were measured at all time points, the number of paired samples available after 12 months of treatment was limited. This can reduce the statistical power and limit the generalisability of the findings. Furthermore, the sample size of 8 patients in pre- and post-transcriptomic analyses could prevent the detection of critical pathways altered in FASD patients due to slight effects in gene expression after EGCG treatment, which could be significant with a larger sample size.

Future research should consider age-stratified analyses to better understand the differential effects of EGCG across FASD. It would be valuable to investigate unexplored facets of EGCG’s effects, such as osteoclast differentiation pathway, and unexplored genes such as IL-1B downregulation present in NLR signaling, by techniques such as Luminex or ELISA. Additionally, because mitochondrial pathways did not reach significance in this dataset, future studies with larger cohorts should include targeted analyses of mitochondrial function to determine whether EGCG may exert effects not detectable in this pilot sample.

## Methods

### Experimental model and participants

Pre-post, open-label, non-randomized pilot study assessed the efficacy of FontUp, a nutritional supplement containing 94% EGCG in improving cognitive performance for children with FASD. FontUp offers longer half-life, greater stability, and lower inter-individual variability than EGCG alone^93^.

The study included 40 patients adopted from EEC, diagnosed with FASD. Participants received EGCG for one year, with blood collection at baseline, 6 and 12 months of treatment for oxidative stress, and transcriptomic analysis. The use of placebo was not permitted in this study because the Ethical Committee deemed it unethical to withhold effective treatment from children, especially for serious conditions. Pediatric trials have stricter regulations on placebos than adult trials, and the Declaration of Helsinki requires that all children receive the best-proven treatment^94,95^. Since prior evidence supported the trial therapy’s efficacy, using a placebo would have been unethical^20,21^.

The recruited pediatric cohorts belonged to previous projects (PI13/01135;OG085818; PI16/00566; PI19/01853). The study was conducted at Hospital del Mar Medical Research Institute and the Hospital Clinic of Barcelona. Enrollment took place from February 2016 to July 2018.

### Clinical trial registration

This study was conducted as part of a registered clinical trial entitled “Epigallocatechin Gallate (EGCG) to Improve Cognitive Performance in Foetal Alcohol Syndrome (FAS) Children (Neuro-SAF)”, registered at ClinicalTrials.gov (Identifier: NCT02558933).

The trial was registered on 22 September 2015, prior to the enrolment of participants, and is publicly accessible through the World Health Organization International Clinical Trials Registry Platform (WHO ICTRP).

### Inclusion and exclusion criteria

Inclusion criteria included adopted subjects (5 to 16 years) from EEC, diagnosed with FASD, residing in Spain. Participants’ guardians consent to supplement administration and to attend all visits. Exclusion criteria included (i) severe neurological disorders or psychiatric conditions, (ii) use of cognitive stimulatory treatment, and (iii) taking antioxidants, vitamins, or green tea.

### Intervention

Patients diagnosed with FASD received 9 mg/kg/day of EGCG through FontUp, a cocoa-flavored nutritional powder provided by the study investigator, a pediatrician specialized in FASD. The supplement was administered at home by caregivers, dissolved in 200 mL of semi-skimmed milk according to written instructions^93^. The 9 mg/kg/day dose is grounded in prior pediatric research^20,21^, and maximum daily limit of 400 mg. FontUp was purchased from Grand Fontaine Laboratories (Barcelona, Spain). Adherence was monitored through caregiver reports and weight assessments were conducted at baseline, 6, and 12 months to adjust dosage. No major deviations from the administration protocol were observed.

FontUp is composed of fats, carbohydrates, proteins, vitamins, minerals, and 94% EGCG (Supplementary Table S6), with these supplementary components specifically designed to improve EGCG’s stability and bioavailability^93^.

Adverse events were assessed at 6- and 12-month visits through structured interviews with caregivers, asking about any changes in behavior, health, or well-being. No severe adverse events or treatment discontinuations were reported. Previous clinical studies have reported that daily doses of 9 mg/kg of EGCG are well-tolerated, without adverse effects on liver or cardiac function^96^.

### Neurocognitive assessment

Wechsler Intelligence Scale for Children (WISC-IV) Fourth Edition^97^ was performed to evaluate cognitive domain. Scores ≤ 70 in three or more domains indicated cognitive delay. Administered at baseline for all participants and 6 and 12 months for FASD. Test-retest was defined in 35 days, and literature declares a 6-months-retest appropriate^98^. Additionally, studies showed that intelligence-scores in FASD remain stable over time^99,100^, making it unlikely that the improvements observed result from natural cognitive development.

Parental report of behavioral concerns was obtained using the Child Behavior Checklist (CBCL)^101^. Scores ≥ 70 were considered significant. Administered at baseline for all participants and 12 months for FASD.

Other neurocognitive measures were obtained from NEPSY-II^102^, covering five different domains: language, memory and learning, social perception, visuospatial processing, and sensorimotor. Delays were defined as scores ≤ 5. Administered to FASD participants at baseline, 6, and 12 months. Test-retest is 21 days, which demonstrates adequate reliability for retesting^103^. Furthermore, studies demonstrated that intelligence-scores remained stable after 8 years in FASD^99^.

### FASD diagnosis

Participants were diagnosed according to 1996 Institute of Medicine (IOM) criteria^104,105^, based on 5 characteristics: (1) confirmed PAE; (2) minor facial abnormalities (thin upper-lip, smooth-philtrum, and short palpebral fissures); (3) growth retardation (height or weight ≤ 10th percentile); (4) brain growth deficiency; and (5) behavioral or cognitive impairments. FAS diagnosis criteria 2, 3, 4, 5 were required. Partial FAS criteria 1, 2, and at least one of criteria 5 or 2, 5, and 3 or 4 were required. Alcohol-related birth defects (ARBD) require the presence of 1 criterion plus 1 structural defect (heart, face, skeleton, kidney, eye, ear, or hands). Alcohol-related neurodevelopmental disorder (ARND) diagnosis required 1 and 5 criteria.

### Blood collection and processing

5 mL of blood was collected in BD Vacutainer SST II Advance (BD Biosciences;367955) at baseline, 6, and 12 months. Blood samples were centrifuged at 1750 g for 10 min at 4 °C immediately after collection to obtain serum. Two 5 mL tubes of blood were collected in BD Vacutainer K3E 15% Aprotinin 250KIU (SGSH; BD361017). Peripheral blood mononuclear cells were isolated by centrifugation in Blood Collection tube BD Vacutainer, BD CPT (Avantor, BDAM362781). RNA was isolated using QIAshredder (Qiagen,79654) and RNAsy Mini-Kit (Qiagen, 74104) protocols. RNA quantification was performed by Nanodrop One (Thermo Fisher Scientific). RNA included had a 260/280 ratio and a 260/260 ratio > 1.8. RNA integrity (RIN > 8) was verified by Agilent 2100 Bioanalyzer before transcriptomic analysis.

### Oxidative stress biomarkers

ROS production was evaluated by measuring lipid-peroxidation metabolites as malondialdehyde (MDA), 8-isoprostane, and enzymatic activity of lactate-dehydrogenase (LDH) in serum at baseline, 6 and 12 months of treatment. MDA was analyzed using lipid peroxidation (MDA) Assay kit (Abcam; ab118970). 8-Isoprostane was evaluated by ELISA (Abcam; ab175819). LDH was measured by Lactate Dehydrogenase Activity Assay Kit (Sigma Aldrich; MAK066). All samples were analyzed in duplicate, and none exceeded the detection or quantification limits.

### RNA sequencing (RNAseq)

RNA was analyzed at Microomics Systems S.L. using an Illumina TruSeq Stranded mRNA, yielding 2 × 125 bp sequences. Quality-control was evaluated using FastQC. Pre-processing with Trimmomatic, alignment to GRCh38 with STAR, and alignment quality-control with FastQC and MultiQC. Reads were counted with featureCounts. Differential expression was analyzed in R (v4.3.1) using DESeq2 with a paired design to compare baseline and 12-month samples. P-values were adjusted using the Benjamini–Hochberg FDR, and genes with FDR < 0.05 were considered significant. Data normalization and Principal Component Analysis (PCA) was achieved with DESeq2 package. Gene annotation was obtained using org.Hs.eg.db package. Enrichment pathway analysis was performed using gage packages.

### RT-qPCR validation

Reverse transcription was performed using Applied Biosystems High Capacity cDNA Reverse Transcription kit (Fisher Scientific;10400745). RT-qPCR was performed using PerfeCTa SYBR Green FastMix (Quantabio;95074-012) in QuantStudioTM 7 (Thermo Fisher Scientific; 4485701). GAPDH served as reference gene. Primers provided by Sigma Aldrich are listed in Supplementary Table S7. The 2-ΔΔCt method assessed relative abundance.

### Study outcomes

The primary outcomes were improvements in cognitive, executive, and behavioral function, assessed using the WISC-IV, NEPSY-II, and CBCL 6–18. Secondary outcomes included oxidative stress biomarkers (MDA, 8-isoprostane, LDH) and molecular changes in gene expression profiles, assessed through RNAseq and validated by RT-qPCR.

### Statistical analysis

Data analysis was performed using SPSS (IBM), GraphPad, and R project. Descriptive statistics included mean and standard deviation (SD). Given the sample size and the non-normal distribution of the data, non-parametric tests were selected. For oxidative stress biomarkers, we performed a paired Wilcoxon signed-rank test comparing baseline vs. 6 months and baseline vs. 12 months. For neurocognitive outcomes, we conducted pre-specified paired comparisons of baseline vs. 6 months and baseline vs. 12 months using the Wilcoxon signed-rank test. To control for multiple testing across these pairwise comparisons, p-values were adjusted using the Holm–Bonferroni procedure (two-sided α = 0.05). Sensitivity analyses (G*Power) indicated that with *n* = 33 pairs the study had 80% power (two-tailed α = 0.05) to detect a minimum effect size of dz = 0.52, and with *n* = 24 pairs the minimum detectable effect was dz = 0.61. Therefore, the study was adequately powered to detect medium-to-large effects, but smaller effects may not have been captured. Post hoc power analysis indicated that, for the neurocognitive outcomes analyzed, the study had sufficient power to detect medium-to-large effects, with *n* = 33 yielding a power of 0.79 (noncentrality parameter = 2.86, df = 30.5, critical t = 2.04) and *n* = 24 yielding a power of 0.79 (noncentrality parameter = 2.92, df = 21.9, critical t = 2.07). These results suggest that the significant improvements observed are robust, although smaller effects may not have been detectable due to the limited sample size. Statistical significance was set at *p* < 0.05. All participants with available data at each time point were included in the analyses. Missing data resulted from non-compliance or inadequate RNA quality. These cases were excluded from the corresponding analyses without data imputation.

## Supplementary Information

Below is the link to the electronic supplementary material.

Supplementary Material 1

Supplementary Material 2

## Data Availability

RNAseq data is available in the Gene Expression Omnibus (GEO) repository under accession number GSE307141. Anonymized neurocognitive and oxidative stress datasets supporting the findings of this study are publicly available in the Zenodo repository, DOI: https://doi.org/10.5281/zenodo.18078285.
