# Supplementary material for: Cognitive and transcriptomic effects of Epigallocatechin gallate in fetal alcohol spectrum disorders
Source: Sci Rep. 2026 Jan 2;16:4461. doi: 10.1038/s41598-025-34576-1 (PMC12864738; doi:10.1038/s41598-025-34576-1)
Supplement: Supplementary file 1 — Supplementary Material 1 [file 41598_2025_34576_MOESM1_ESM.docx]

SUPPLEMENTARY FIGURES


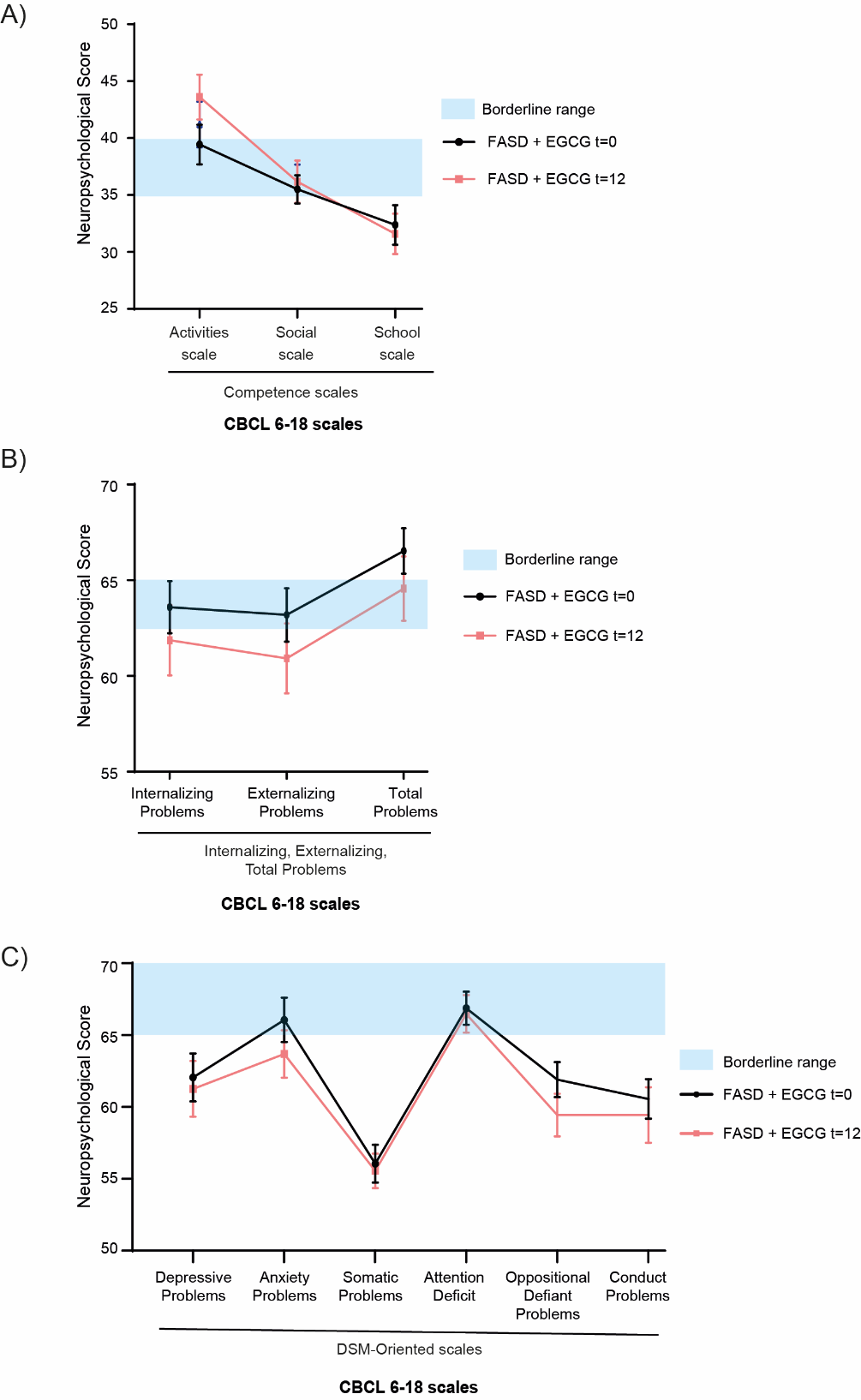


**Figure S1. CBCL 6-18 Neurocognitive assessment.** Comparison of CBCL profiles of FASD at baseline and 12 months after EGCG treatment. A) Competence scales. B) Internalizing, Externalizing, Total Problems. C) DSM-Oriented scales. The graph represents the mean ± SEM of each score. Blue line represents distribution of control patients, black line represents distribution of FASD patients at baseline, and red line represents patients after 12 months of EGCG treatment.


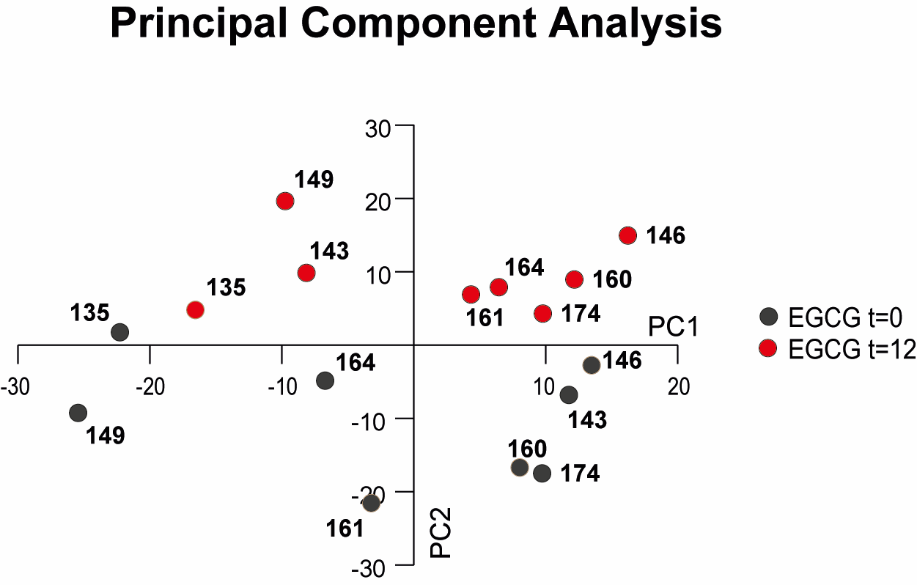


**Figure S2.** Principal Component Analysis (PCA). Black dots indicate baseline and red dots indicate 12 months after EGCG treatment.
