# Supplementary material for: Cognitive and transcriptomic effects of Epigallocatechin gallate in fetal alcohol spectrum disorders
Source: Sci Rep. 2026 Jan 2;16:4461. doi: 10.1038/s41598-025-34576-1 (PMC12864738; doi:10.1038/s41598-025-34576-1)
Supplement: Supplementary file 2 — Supplementary Material 2 [file 41598_2025_34576_MOESM2_ESM.docx]

**Supplementary Tables**

**Table S1**. WISC-IV neurocognitive assessment. Non-parametric *Wilcoxon* signed-rank test was performed to compare the scores of FASD children at baseline (0), after 6 (6m) and 12 (12m) months of EGCG treatment. Multiple pairwise comparisons were corrected using Holm–Bonferroni adjustment.

| **WISC-IV** | **Scores (Mean ± SEM)** | | | ***P-value; Wilcoxon*** | |
| --- | --- | --- | --- | --- | --- |
|  | **0** | **6m** | **12m** | **0 vs 6m** | **0 vs 12m** |
|  | (n=40) | (n=33) | (n=24) |  |  |
| FSIQ | 73.8±3.1 | 71.8±3.2 | 72.0±3.3 | 1.00 | 1.00 |
| VCI | 72.0±2.5 | 72.5±2.5 | 74.2±3.1 | 1.00 | 1.00 |
| PRI | 75.3±2.9 | 78.9±2.8 | 82.9±3.2 | 0.18 | **0.002*** |
| WMI | 71.8±2.7 | 76.3±3.1 | 77.5±3.9 | 0.19 | **0.04*** |
| PSI | 82.1±3.3 | 83.2±3.5 | 84.6±3.2 | 0.78 | 0.78 |

Abbreviations: FSIQ: full-scale intelligence quotient; m: month; PRI: perceptual reasoning index; PSI: processing speed index; SEM: Standard Error Mean; VCI: verbal comprehension index; WMI: working memory index. P-values (Wilcoxon signed-rank test) were adjusted for multiple testing using the Holm–Bonferroni method. * and bold for significance p-value<0.05.

**Table S2**. CBCL Neurocognitive assessment. Non-parametric Wilcoxon signed-rank tests for related samples were performed to compare the scores of FASD children at baseline (0) and 12 months (12m) of EGCG treatment.

| ***Neurocognitive assessment*** | **Scores (Mean ± SEM)** | | ***P-value; Wilcoxon*** |
| --- | --- | --- | --- |
| ***CBCL*** | **0m** | **12m** | **0 *vs* 12m** |
|  | (n=40) | (n=24) |  |
| **Competence** |  |  |  |
| Activities | 39.4±1.7 | 43.6±2.0 | 0.38 |
| Social | 35.5±1.2 | 36.2±1.9 | 0.84 |
| School | 32.4±1.7 | 31.6±1.8 | 0.9 |
| **Syndrome** |  |  |  |
| Anxious/depressed | 63.8±1.6 | 62.1±1.7 | 0.9 |
| Withdrawn/depressed | 63.7±1.7 | 63.2±2.0 | 0.8 |
| Somatic Complaints | 57.4±1.4 | 57.3±1.4 | 0.89 |
| Social Problems | 67.2±1.3 | 66.9±1.8 | 0.97 |
| Thought Problems | 63.7±1.7 | 62.3±1.8 | 0.95 |
| Attention Problems | 70.1±1.5 | 70.0±1.5 | 0.99 |
| Rule-Breaking behavior | 60.1±1.4 | 59.4±1.6 | 0.68 |
| Aggressive behavior | 65.4±1.5 | 62.3±1.9 | **0.03*** |
| **Internalizing, Externalizing, Total Problems** | | | |
| Internalizing Problems | 63.6±1.4 | 61.9±1.8 | 0.51 |
| Externalizing Problems | 63.2±1.4 | 61.0±1.8 | 0.08 |
| Total Problems | 66.5±1.2 | 64.6±1.7 | 0.18 |
| ***DSM-Oriented scores*** |  |  |  |
| Depressive Problems | 62.1±1.7 | 61.3±1.9 | 0.98 |
| Anxiety Problems | 66.1±1.5 | 63.7±1.7 | 0.6 |
| Somatic Problems | 56.1±1.3 | 55.6±1.2 | 0.97 |
| Attention Deficit | 66.9±1.1 | 66.5±1.3 | 0.98 |
| Oppositional Defiant Problems | 61.9±1.2 | 59.4±1.4 | 0.12 |
| Conduct Problems | 60.5±1.4 | 59.4±1.9 | 0.52 |

Abbreviations: m: month; SEM: standard error mean. * and bold for significance p-value<0.05.

**Table S3**. **NEPSY-II Neurocognitive assessment.** Non-parametric Wilcoxon signed-rank tests for related samples were performed to compare the scores of FASD children at baseline (0), after 6 (6m) and 12 (12m) months of EGCG treatment. Multiple pairwise comparisons were corrected using Holm–Bonferroni adjustment.

| **Neurocognitive assessment** | **Scores (Mean ±SEM)** | | | **P-value; Wilcoxon** | |
| --- | --- | --- | --- | --- | --- |
| **NEPSY-II** | **0** | **6m** | **12m** | **0 vs 6m** | **0 vs 12m** |
|  | (n=40) | (n=33) | (n=24) |  |  |
| **Language** |  |  |  |  |  |
| Comprehension of Instructions (CI) | 3.9±0.5 | 4.4±0.7 | 4.3±0.6 | 1.00 | 1.00 |
| Semantic Word Generation (SWG) | 5.9±0.6 | 6.5±0.8 | 6.8±0.7 | 0.91 | 0.22 |
| Initial Word Generation (IWG) | 5.6±0.5 | 5.8±0.6 | 5.3±0.6 | 0.84 | 0.21 |
| **Memory and Learning** |  |  |  |  |  |
| Memory for Faces (MF) | 7.3±0.7 | 9.1±0.5 | 10.0±0.8 | **0.02*** | **0.005*** |
| Memory for Faces Delayed (MFD) | 6.5±0.7 | 9.1±0.5 | 9.1±0.8 | **0.003*** | **0.006*** |
| List Memory & List Memory Delayed (LM&LMD) | 4.0±0.5 | 4.9±0.7 | 5.1±1.0 | 0.09 | 0.09 |
| Narrative Memory (NM) | 6.0±0.6 | 6.2±0.8 | 5.0±0.6 | 0.74 | 0.17 |
| **Social Perception** |  |  |  |  |  |
| Affect Recognition (AR) | 6.8±0.7 | 6.6±0.5 | 7.4±0.7 | 1.00 | 1.00 |
| Theory of Mind (TM) | 3.1±0.4 | 3.3±0.4 | 3.1±0.3 | 0.6 | 1.00 |
| **Visuospatial Processing** |  |  |  |  |  |
| Design Copying (DC) | 4.1±0.6 | 3.5±0.6 | 5.4±2.0 | 1.00 | 0.75 |
| **Sensorimotor functions** |  |  |  |  |  |
| Visuomotor Precision Time (VPTS) | 10.6±0.9 | 9.2±1.1 | 8.4±1.9 | 0.08 | 0.08 |
| Visuomotor Precision Combined (VPC) | 8.7±0.8 | 9.4±0.8 | 6.6±1.5 | 0.78 | 0.27 |

Abbreviations: m: month; SEM: standard error mean. P-values (Wilcoxon signed-rank test) were adjusted for multiple testing using the Holm–Bonferroni method. * *p-value* < 0.05.

**Table S4.** KEGG pathways list represented with the significant genes comparing baseline and treatment with EGCG at 12 months (p<0.05). Grey represents not significant pathways.

| KEGG pathways | p.val |
| --- | --- |
| hsa04062 Chemokine signaling pathway | 0.0030 |
| hsa04612 Antigen processing and presentation | 0.0049 |
| hsa04145 Phagosome | 0.0080 |
| hsa04621 NOD-like receptor signaling pathway | 0.0117 |
| hsa04975 Fat digestion and absorption | 0.0123 |
| hsa04010 MAPK signaling pathway | 0.0150 |
| hsa04146 Peroxisome | 0.0222 |
| hsa04380 Osteoclast differentiation | 0.0253 |
| hsa03013 RNA transport | 0.0265 |
| hsa03320 PPAR signaling pathway | 0.0292 |
| hsa00520 Amino sugar and nucleotide sugar metabolism | 0.0299 |
| hsa04144 Endocytosis | 0.0327 |
| hsa03040 Spliceosome | 0.0408 |
| hsa04141 Protein processing in endoplasmic reticulum | 0.0456 |
| hsa04623 Cytosolic DNA-sensing pathway | 0.0477 |
| hsa04666 Fc gamma R-mediated phagocytosis | 0.0519 |
| hsa00280 Valine, leucine and isoleucine degradation | 0.0536 |
| hsa00240 Pyrimidine metabolism | 0.0566 |
| hsa03410 Base excision repair | 0.0571 |
| hsa03050 Proteasome | 0.0585 |
| hsa04620 Toll-like receptor signaling pathway | 0.0587 |
| hsa00564 Glycerophospholipid metabolism | 0.0668 |
| hsa00532 Glycosaminoglycan biosynthesis - chondroitin sulfate | 0.0676 |
| hsa04114 Oocyte meiosis | 0.0688 |
| hsa04340 Hedgehog signaling pathway | 0.0806 |
| hsa04650 Natural killer cell mediated cytotoxicity | 0.0815 |
| hsa04916 Melanogenesis | 0.0821 |
| hsa03020 RNA polymerase | 0.0869 |
| hsa04210 Apoptosis | 0.0884 |
| hsa04310 Wnt signaling pathway | 0.0908 |
| hsa04640 Hematopoietic cell lineage | 0.0938 |
| hsa04330 Notch signaling pathway | 0.0957 |

**Table S5.** Biomarkers of hepatic function and metabolic risk at baseline and after 12 months of treatment. Data were compared using paired statistical tests (paired t-test or Wilcoxon signed-rank test).

|  | **Baseline**  **Mean±SD**  **(n=12)** | **12 months**  **Mean±SD**  **(n=12)** | **Changes from**  **baseline** | **p-value** |
| --- | --- | --- | --- | --- |
| Alanine aminotransferase  (ALT) (UI/L) | 12.94 (5.47) | 14.22 (4.67) | 1.28 | 0.068 |
| Aspartate aminotransferase  (AST) (UI/L) | 24.50 (4.59) | 24.39 (4.45) | -0.11 | 0.913 |
| Glucose (mg/dL) | 92.11 (14.53) | 88.28 (11.31) | -3.83 | 0.417 |
| Cholesterol (mg/dL) | 158.61 (25.86) | 156.78 (30.24) | -1.83 | 0.715 |
| Triglycerides (mg/dL) | 91.22 (45.23) | 55.00 (21.39) | -36.22 | **0.0036** |

**Table S6.** Food supplement FontUp composition

| **Average analysis** | **Per 100 mL** | **Per ration** |
| --- | --- | --- |
| **Energetic value (kJ/kcal)** | 1606/383 | 787/188 |
| **Fats (g)** | 11 | 5.4 |
| - **Saturated (g)** | 4.4 | 2.2 |
| - **Monounsaturated (g)** | 4.4 | 2.2 |
| - **Polyunsaturated (g)** | 2.2 | 1.1 |
| - **DHA (mg)** | 5.4 | 2.6 |
| **Carbohydrates (g)** | 47 | 23 |
| - **Dietary fibre (g)** | 16 | 7.9 |
| - **Fructo-ologosaccharides (g)** | 3.1 | 1.5 |
| **Proteins (g)** | 16 | 8 |
| **Salt (g)** | 0.52 | 0.3 |
| **Minerals** | | |
| **Na (mg)** | 206 | 101 |
| **K (mg)** | 606 | 297 |
| **Cl (mg)** | 395 | 194 |
| **Ca (mg)** | 221 | 108 |
| **P (mg)** | 209 | 102 |
| **Mg (mg)** | 106 | 52 |
| **Fe (mg)** | 4.6 | 2.3 |
| **Zn (mg)** | 3.1 | 1.5 |
| **Cu (μg)** | 0.5 | 0.2 |
| **Mn (mg)** | 0.5 | 0.2 |
| **F (μg)** | 0.1 | 0.05 |
| **Se (μg)** | 10 | 4.9 |
| **Cr (μg)** | 16 | 7.8 |
| **Mo (μg)** | 10 | 4.9 |
| **I (μg)** | 21 | 10 |
| **Vitamins** | | |
| **Vitamin A (μg)** | 251 | 123 |
| **Vitamin D (μg)** | 1.9 | 0.9 |
| **Vitamin E (mg)** | 4.2 | 2.1 |
| **Vitamin K (μg)** | 15.0 | 7.4 |
| **Vitamin C (mg)** | 25 | 12 |
| **Thiamine (B1) (mg)** | 0.3 | 0.1 |
| **Riboflavin (B2) (mg)** | 0.4 | 0.2 |
| **Niacin (B3/PP) (mg)** | 4.0 | 2.0 |
| **Vitamin B6 (mg)** | 0.4 | 0.2 |
| **Folic acid (B9) (μg)** | 80.0 | 39 |
| **Vitamin B12 (μg)** | 0.5 | 0.2 |
| **Biotin (μg)** | 58.0 | 28 |
| **Pantothenic acid (B5) (mg)** | 1.9 | 0.9 |
| **Others** | | |
| **Green tea extract (mg) (minimum 250 mg EGCG per ration)** | 543 | 266 |
| **Osmolarity (mOsm/L)** | 635 | |

**Table S7.** Primer sequences used for real-time quantitative reverse transcriptase polymerase chain reaction (RT-qPCR).

| **Gene** | **5' to 3'** | **Sequence** | **Amplicon length (bp)** | **Efficiency (%)** |
| --- | --- | --- | --- | --- |
| FOS | Sense | AGAATCCGAAGGGAAAGGAA | 150 | 97 |
| FOS | Anti-Sense | CTTCTCCTTCAGCAGGTTGG |  |  |
| CX3CR1 | Sense | GTGGTGCTGACAAAGCTTGGAA | 94 | 93 |
| CX3CR1 | Anti-Sense | TCACTGGGTGCCATCGTAAGAA |  |  |
| CXCR3 | Sense | GCCCTCTACAGCCTCCTCTT | 119 | 93 |
| CXCR3 | Anti-Sense | ACAGCTAGGTGGAGCAGGAA |  |  |
| CCR2 | Sense | AGAGGCATAGGGCAGTGAGA | 141 | 97 |
| CCR2 | Anti-Sense | CCAGTTGACTGGTGCTTTCA |  |  |
| CASP5 | Sense | AAAGAACAACGTGGCTGGAC | 126 | 110 |
| CASP5 | Anti-Sense | TTGCCCAGGTATTCCAACAT |  |  |
| TNFAIP3 | Sense | GCTGGCAACTGGAGTCTCTC | 122 | 110 |
| TNFAIP3 | Anti-Sense | CATGGGTGTGTCTGTGGAAG |  |  |
| FGF11 | Sense | CTCATCCTGCTGTCCAAGGT | 102 | 93 |
| FGF11 | Anti-Sense | GCAGAACAGTTTGGTGACGA |  |  |
| DUSP4 | Sense | TCACGGCTCTGTTGAATGTC | 100 | 105 |
| DUSP4 | Anti-Sense | ATGTCGGCCTTGTGGTTATC |  |  |
| ABCD2 | Sense | TATGTTTGGAGCAGCAGTGG | 131 | 105 |
| ABCD2 | Anti-Sense | CGAGCAGTGGTAAAGGCTTC |  |  |
| PMVK | Sense | CGGAGAGTGTCTGACATCCA | 137 | 110 |
| PMVK | Anti-Sense | TCTGACTCAGCATCGTCCAC |  |  |
| OLR1 | Sense | CCTATTTTCCTCGGGCTCAT | 122 | 101 |
| OLR1 | Anti-Sense | AGGAAATTGCTTGCTGGATG |  |  |
